# A consensus statement on birth defects surveillance, prevention, and care in Latin America and the Caribbean

**DOI:** 10.26633/RPSP.2019.2

**Published:** 2019-02-14

**Authors:** Ignacio Zarante, Paula Hurtado-Villa, Salimah R. Walani, Vijaya Kancherla, Jorge López Camelo, Roberto Giugliani, Boris Groisman, Christopher P. Howson, Pablo Durán

**Affiliations:** 1 Pontificia Universidad Javeriana Pontificia Universidad Javeriana School of Medicine Human Genetics Institute Bogotá Colombia Human Genetics Institute, School of Medicine, Pontificia Universidad Javeriana, Bogotá, Colombia.; 2 Pontificia Universidad Javeriana Pontificia Universidad Javeriana Faculty of Health Sciences Basic Health Sciences Department Cali Colombia Basic Health Sciences Department, Faculty of Health Sciences, Pontificia Universidad Javeriana, Cali, Colombia.; 3 March of Dimes March of Dimes ArlingtonVirginia United States of America March of Dimes, Arlington, Virginia, United States of America.; 4 Emory University Rollins School of Public Health Department of Epidemiology Center for Spina Bifida Prevention AtlantaGeorgia United States of America Center for Spina Bifida Prevention, Department of Epidemiology, Rollins School of Public Health, Emory University, Atlanta, Georgia, United States of America.; 5 Center for Medical Education and Clinical Research (CEMIC-CONICET) Center for Medical Education and Clinical Research (CEMIC-CONICET) Latin American Collaborative Study of Congenital Malformations (ECLAMC) Buenos Aires Argentina Latin American Collaborative Study of Congenital Malformations (ECLAMC), Center for Medical Education and Clinical Research (CEMIC-CONICET), Buenos Aires, Argentina.; 6 Hospital de Clinicas de Porto Alegre Federal University of Rio Grande do Sul Medical Genetics Service Department of Genetics Porto Alegre Brasil Department of Genetics, Federal University of Rio Grande do Sul, Medical Genetics Service, Hospital de Clinicas de Porto Alegre, Porto Alegre, Brazil.; 7 National Administration of Laboratories and Health Institutes National Administration of Laboratories and Health Institutes National Center of Medical Genetics National Network of Congenital Anomalies of Argentina Buenos Aires Argentina National Network of Congenital Anomalies of Argentina, National Center of Medical Genetics, National Administration of Laboratories and Health Institutes, Buenos Aires, Argentina.; 8 Howson & Partners for Global Health Howson & Partners for Global Health Santa FeNew Mexico United States of America Howson & Partners for Global Health, Santa Fe, New Mexico, United States of America.; 9 Pan American Health Organization/World Health Organization Pan American Health Organization/World Health Organization Women’s and Reproductive Health Latin American Center for Perinatology Montevideo Uruguay Latin American Center for Perinatology, Women’s and Reproductive Health, Pan American Health Organization/World Health Organization, Montevideo, Uruguay.

**Keywords:** Congenital abnormalities, epidemiology, medical care, neonatal screening, prenatal diagnosis, primary prevention, surveillance, Latin America, West Indies, Anomalías congénitas, epidemiología, atención médica, tamizaje neonatal, diagnóstico prenatal, prevención primaria, vigilancia, América Latina, Indias Occidentales, Anormalidades congênitas, epidemiologia, cuidados médicos, triagem neonatal, diagnóstico pré-natal, prevenção primária, vigilância, América Latina, Índias Ocidentais

## Abstract

Birth defects contribute up to 21% of the mortality in those under 5 years of age in Latin America and the Caribbean (LAC), and that burden has been compounded by the Zika virus epidemic. In 2001, the March of Dimes launched a series of biennial assemblies called the International Conference on Birth Defects and Disabilities in the Developing World (ICBD). The latest ICBD, in 2017, convened in Bogotá, Colombia, and was attended by over 300 professionals, policymakers, and donors. The conference attendees, a majority of whom were from LAC, supported a call to action in the form of a consensus statement. The consensus statement lists key actions for maximizing birth defects surveillance, prevention, and care in LAC: 1) improving surveillance; 2) reducing risks for birth defects; 3) fortifying staple foods; 4) preventing and treating infections associated with birth defects; 5) implementing newborn screening; 6) providing care and services for people with birth defects and disabilities; 7) involving governments, civil society, and international agencies; and 8) advancing research for birth defects. Implementation and scale-up of evidence-based interventions using multisectoral and multidisciplinary collaborative approaches were endorsed. LAC countries can leverage technology and social media to advance and advocate for approaches identified in the consensus statement. The consensus statement can be used as a guide by both governments and nongovernmental agencies to take immediate steps for improving the quality of life of those living with birth defects and associated disabilities in the LAC countries.

## RESUMEN

Los defectos congénitos contribuyen hasta con el 21% de la mortalidad de los menores de 5 años en América Latina y el Caribe (ALC); la epidemia de síndrome congénito por el virus del Zika aumentó esa carga. En el 2001, la organización March of Dimes instituyó una serie de asambleas bienales denominadas Conferencia internacional sobre defectos congénitos y discapacidades en el mundo en desarrollo (ICBD). La más re-ciente, en el 2017, se llevó a cabo en Bogotá (Colombia) y contó con la presencia de más de 300 profesionales, responsables de las políticas y donantes. Los asistentes a la conferencia, en su mayoría de América Latina y el Caribe, apoyaron un llamamiento a la acción en forma de una declaración de consenso. Dicha declaración enumera las acciones clave para potenciar al máximo la vigilancia, la prevención y la atención de los defectos congénitos en América Latina y el Caribe, a saber: 1) mejorar la vigilancia; 2) reducir los factores de riesgo asociados a defectos congénitos; 3) fortificar los alimentos de primera necesidad; 4) prevenir y tratar las infecciones asociadas con los defectos congénitos; 5) instituir programas de tamizaje a los recién nacidos; 6) prestar atención y servicios a las personas con defectos congénitos y discapacidades; 7) hacer participar a los gobiernos, la sociedad civil y los organismos internacionales; y 8) promover la investigación sobre los defectos congénitos. Se respaldaron la ejecución y la ampliación a mayor escala de las intervenciones basadas en evidencia, con enfoques colaborativos multisectoriales y multidisciplinarios. Los países de América Latina y el Caribe pueden aprovechar la tecnología y las redes sociales para impulsar y promover las estrategias mencionadas en la declaración de consenso. Por su parte, tanto los gobiernos como los organismos no gubernamentales pueden usar la declaración de consenso como una guía para adoptar medidas inmediatas para mejorar la calidad de vida de las personas con defectos congénitos y las discapacidades asociadas, en los países de América Latina y el Caribe.

## RESUMO

As malformações congênitas constituem 21% das causas de morte em crianças menores de 5 anos na América Latina e no Caribe (ALC) e este ônus tem sido agravado pela epidemia do vírus zika. Em 2001, a March of Dimes lançou uma série de encontros bienais denominada International Conference on Birth Defects and Disabilities in the Developing World (conferência internacional sobre malformações congênitas e deficiências no mundo em desenvolvimento, ICBD). A última ICBD foi realizada em Bogotá, na Colômbia, em 2017 e contou com a participação de mais de 300 profissionais, formuladores de políticas e doadores. Os participantes da conferência, na sua maioria da ALC, demonstraram apoio a um chamado à ação na forma de uma declaração de consenso. Nela, enumeram-se as principais ações para aumentar ao máximo a vigilância, prevenção e atenção às malformações congênitas na ALC: 1) melhorar a vigilância; 2) reduzir os riscos de malformações congênitas; 3) fortificar os gêneros alimentícios de primeira necessidade; 4) prevenir e tratar as infecções associadas às malformações congênitas; 5) implementar testes de detecção em recém-nascidos; 6) prestar atendimento e serviços aos portadores de malformações congênitas e deficiências; 7) atrair a participação de governos, sociedade civil e organismos internacionais e 8) incentivar o progresso da pesquisa na área de malformações congênitas. Foram endossadas a implementação e a expansão das intervenções com fundamentação científica usando enfoques colaborativos multissetoriais e multidisciplinares. Os países da ALC devem se valer de tecnologia e das mídias sociais para promover e defender os enfoques identificados na declaração de consenso. A declaração pode servir como guia aos governos e organismos não governamentais ao tomarem medidas imediatas para melhorar a qualidade de vida de quem vive com malformações congênitas e deficiências associadas nos países da ALC.

Birth defects contribute up to 21% of under-5 mortality in Latin America and the Caribbean (LAC), compared to a global average of 9% (1). The burden has been further compounded by the recent Zika virus epidemic in LAC (2, 3). The United Nations Development Program (UNDP) and its partners estimated the social and economic impact of Zika alone in LAC would be between US$ 7 billion and US$ 18 billion over the period 2015–2017 (4). The cost of inaction on birth defects for individuals, families, and the society at large cannot be ignored.

At the 63^rd^ World Health Assembly, in 2010, a resolution on birth defects was passed to address widespread concerns related to lack of recognition of the global burden of birth defects and the limited resources for prevention and management of birth defects (5). The resolution urged Member States of the World Health Organization (WHO) to take steps in prioritizing birth defects surveillance, prevention, and care. It called on governments, civil society, and the public to raise awareness about the importance of birth defects, and to mobilize policy and programs to reduce morbidity and mortality associated with birth defects by integrating birth defects into existing maternal, reproductive, and child health services. Additionally, emphasis was placed on the social welfare of all individuals with birth defects.

Efforts on birth defects have also recently been energized with the launch of the Sustainable Development Goals (SDGs) (6). Given the burden of birth defects and disability in LAC, it is not possible to meet SDG 3.2 (“by 2030, end preventable deaths of newborns and children under 5 years of age, with all countries aiming to reduce neonatal mortality to at least as low as 12 per 1 000 live births and under-5 mortality to at least as low as 25 per 1 000 live births”) and SDG 4.2.1 (“proportion of children under 5 years of age who are developmentally on track in health, learning and psychosocial well-being, by sex”) without addressing the birth defects and related disability. In alignment with SDG 3 of health for all, there is a need for immediate action to maximize birth defects surveillance, prevention, and care in LAC, so as to increase the chances for each pregnancy to have a healthy outcome and for an improved quality of life for populations affected by birth defects and related disabilities.

In 2001, recognizing the need to build capacity in lower-income countries for the prevention of birth defects and care of those affected, the March of Dimes (an organization in the United States of America that promotes the health of all mothers and babies worldwide (www.marchofdimes.org)), launched a series of biennial assemblies called the International Conference on Birth Defects and Disabilities in the Developing World (ICBD). The goal of these events has been to provide a platform for the sharing of resources and knowledge to strengthen surveillance and health care delivery for prevention and care, to influence public health policy, and to mobilize funding for birth defects in countries worldwide. The latest ICBD, in 2017 in Bogotá, Colombia, was attended by over 300 professionals, policymakers, and donors from 31 countries worldwide (7). About 60% of the participants were from LAC.

The participants at the ICBD 2017 conference agreed that there was a need to publish a consensus statement on birth defects specific to the LAC countries. In addition, the conference proceedings highlighted the need to consider the cost-effectiveness and the cost-benefit of the proposed actions. Overall, there was a general agreement to the call to action described below for improving surveillance and prevention of birth defects, increasing access to quality medical care and services, and enhancing the role of the civil society in advocating for an increased quality of life for those affected by birth defects.

A panel of 15 individuals with birth defects expertise in LAC, and who were also present at the ICBD 2017, provided input for developing a consensus statement specific to LAC. The authors then shared the original concepts that formed the content of the consensus statement with the conference participants, seeking their endorsement. A total of 130 participants endorsed the original concepts. The majority of those who endorsed the concepts were from LAC countries, including Argentina, Brazil, Colombia, Costa Rica, Ecuador, and Uruguay. The following call to action is for maximizing birth defects surveillance, prevention, and care in LAC. The call to action is followed by a discussion section that provides background on and describes the importance of the 2017 consensus statement.

## THE CALL TO ACTION

### Improving surveillance

1. Ensuring consistency in terminology for defining, coding, and classifying birth defects; standardizing data collection tools and processes; assuring data integrity through quality management, training, monitoring, and evaluation; and forging collaborations between surveillance systems for timely identification and coordinated response to epidemics.
2. Expanding surveillance systems to increase geographic and population coverage while ensuring that functional or developmental defects are also counted along with structural birth defects.
3. Integrating technologies such as mobile applications, data visualization, and geographic information systems to improve surveillance efficiency.
4. Extending surveillance programs to survey prevalence of risk factors in women of childbearing age (e.g., nutrition, infectious disease control) and birth defects–related disability and mortality in the population.
5. Prioritizing birth defects surveillance as a part of national or regional health systems that are backed by ministries of health.

### Reducing risk for birth defects

1. Raising public health awareness about evidence-based actions individuals and families can take to reduce their risks of having a child with birth defects. Health information should be culturally relevant and be accessible to all women regardless of their geographic location, education level, and socioeconomic status.
2. Educating women and girls, and men and boys, about their risks in a variety of settings and using different approaches. Schools, workplaces, and community centers can serve as places for delivering health education programs. For a population-based approach, use of social media, Web, and mobile technologies and platforms for popular culture are essential and innovative tools.
3. Maximizing the health of women and adolescent girls not only during pregnancy, but also aiming at the critical preconception period, by focusing on exposures to teratogenic substances (e.g., tobacco, alcohol, recreational drugs, and medications), primary prevention, screening and treatment of infections, ensuring optimal nutrition, immunization and vector control, increasing access to family planning and contraception, and reducing environmental exposures and pollution.
4. Training health professionals in assessing and addressing risk factors for birth defects in a primary care setting.
5. Empowering women with health knowledge so that they can seek care and services to reduce their risks.

### Fortifying staple foods

1. Implementing mandatory food fortification of staples with at least iron and folic acid in all countries in LAC.
2. Ensuring quality control of fortification standards through periodic monitoring of fortified foods in countries with existing food fortification programs. In addition, effectiveness of fortification should be evaluated through population-based biomarker analysis.

### Preventing and treating infections

1. Investing in developing population-based infection control programs and policies (e.g., health education, immunizations, disease vector control, sanitation, sexual transmitted infections screening) through sustainable funding and resources.
2. Building laboratory and clinical care capacity for timely detection of infections through serological or other confirmatory tests.
3. Improving access to infection prevention and treatment through primary health care programs and referral chains and ensuring coverage for everyone, regardless of their ability to pay.

### Implementing newborn screening

1. Mandating newborn screening programs, supported by resource allocation for training and capacity-building, and monitoring for quality and cost-effectiveness.
2. Developing centralized mechanisms for screening using standardized approaches and promoting shared laboratory capacity at national and regional levels.
3. Improving the coverage of newborn screening to reach everyone, and ensuring inclusion of essential conditions in the screening panel, while also making sure that timely follow-up and treatment are available based on the diagnosis.

### Providing care and services for people with birth defects and disabilities

1. Establishing a holistic approach that is multidisciplinary and multisectoral, adequately meeting health, educational, occupational, rehabilitation, and social needs of those with birth defects and disabilities throughout their life course, while increasing the number of trained professionals who can provide the aforementioned services.
2. Ensuring timely treatment, including surgery, medications, and nutrition, and providing universal coverage to those in need. In addition, home- and community-based follow-up is essential to maximize health and well-being.

### Involving governments, civil society, and international agencies

1. Governments should support primary prevention where applicable and ensure integrated, population-based care programs. Policies, allocation of resources, and monitoring for quality and efficiency are essential parts of the governments’ role in reducing inequalities. Policies and programs should be sustained through relevant ministries within the governments, irrespective of changing political and economic environments.
2. Civil society should actively engage in raising awareness and advocate for reducing disparity, stigma, and isolation of individuals and families affected by birth defects and disabilities. Civil society, including parents, funders, and nongovernmental organizations, should hold governments accountable to provide universal access to care and services.
3. International agencies such as the United Nations Children’s Fund (UNICEF), WHO, and the Pan American Health Organization (PAHO) should integrate and address birth defects. This will influence the governments to accelerate efforts that will help individuals with birth defects to survive, thrive, and transform their futures.

### Advancing research on birth defects

1. Advancing current knowledge about the causes, diagnostics, and treatment modalities through basic research.
2. Supporting epidemiologic research to better understand the role of factors such as sociodemographic, behavioral, nutritional, environmental, and genetic ones, as well as their interactions, in influencing the risk of birth defects.
3. Conducting implementation research on interventions for birth defects prevention and care in the population, and measuring the outcomes and impact of these interventions for a scale-up.
4. Ensuring sustained funding, training, and mentoring of researcher, multidisciplinary, and multicountry collaborations that will support the aforementioned research activities in LAC.

## DISCUSSION

This consensus statement from the 8th ICBD in 2017 is specific to Latin America and the Caribbean, and it is in alignment with the road map of the Global Strategy for Women’s, Children’s and Adolescents’ Health (2016–2030) (8), as part of the Every Women Every Child global movement, and under the auspices of the United Nations SDG 3 (6). In addition, it builds on a global consensus statement from the 2015 Dar es Salaam 7^th^ ICBD. The Dar es Salaam statement recognized that children with birth defects remain left behind in policies, programs, research, and funding, and that there is little to no action in a positive direction to address these gaps with birth defects (9). The 2017 consensus statement for LAC provides specific recommendations for birth defects prevention and care, while taking into account the region’s tremendous progress and unique challenges that still remain (10-15). The consensus is driven by the impact of the recent Zika virus epidemic on microcephaly and the needs of those affected (16-18), while recognizing the current status and advances made in LAC on birth defects surveillance through integrated approaches. Some examples of such advances include the Latin American Collaborative Study of Congenital Malformations (ECLAMC) (19) and PAHO’s successful role in promoting mandatory food fortification policies (20). The 2017 statement, however, takes into consideration the gaps that persist in the LAC region with regards to surveillance, diagnosis, research, access to care, program coverage, monitoring, and evaluation for those with birth defects (21-23).

Recently developed concepts in triple surveillance for integrated strategies to support and accelerate birth defect prevention (24) can be implemented in LAC utilizing existing surveillance infrastructure in the region. Although there is diversity in LAC in terms of geopolitical environments, health systems, and economies, most countries in the region share a common language and similar cultural contexts, which can be advantageous for developing regional collaborations and sharing resources. Implementation and scale-up of evidence-based interventions using multisectoral and multidisciplinary collaborative approaches can be of benefit to populations across the region. Furthermore, LAC has high information and communication technology utilization (25), and so must leverage technology and social media to advance and advocate for the approaches identified in this consensus statement.

## Conclusion

Through this consensus statement, the authors and the endorsing attendees of the 2017 ICBD call upon governments, nongovernmental and civil society organizations, health care service organizations, institutions of higher education, donor agencies, researchers, and other stakeholders to take actions to improve birth defects prevention and care, as well as the quality of life of those living with birth defects and associated disabilities in Latin America and the Caribbean. We also call on civil society in the LAC countries to demand accountability from their governments and other relevant stakeholders.

## Author contributions.

SRW and CPH conceived the original idea. IZ, PHV, SRW, and VK collected data, interpreted the results, and wrote the first draft. JLC, RG, BG, CPH, and PD contributed to the development of the paper. All the authors reviewed and approved the final version.
